# The Effect of Steroid and Mannitol Combination Treatment on Postoperative Rehabilitation of Multiple Metacarpal Bone Fractures

**DOI:** 10.3390/medicina59040783

**Published:** 2023-04-17

**Authors:** Jangyoun Choi, Hye Jin Seo, Jongweon Shin, Jun-Hee Byun, Sung No Jung

**Affiliations:** 1 Department of Plastic and Reconstructive Surgery, Seoul St. Mary’s Hospital, College of Medicine, The Catholic University of Korea, Seoul 06591, Republic of Korea; 2 Department of Plastic and Reconstructive Surgery, Eunpyeong St. Mary’s Hospital, College of Medicine, The Catholic University of Korea, Seoul 03312, Republic of Korea; 3 Department of Plastic and Reconstructive Surgery, Uijeongbu St. Mary’s Hospital, College of Medicine, The Catholic University of Korea, Seoul 11765, Republic of Korea

**Keywords:** steroid, mannitol, hand rehabilitation, hand fracture, metacarpal bone

## Abstract

*Background and Objectives:* The expedient resolution of postoperative soft tissue edema is particularly important in hand surgery. Prolonged edema and pain become an obstacle to postoperative rehabilitation, delay return to daily life, and in severe cases, lead to a permanent decrease in range of motion. Based on the common physiology between postoperative hand swelling and complex regional pain syndrome (CRPS), we sought to determine if postoperative mannitol and steroid administration to multiple metacarpal bone fracture patients effectively reduces hand swelling and pain and is beneficial for hand rehabilitation. *Materials and Methods:* From March 2015 to February 2019, 21 patients who received closed pinning for multiple metacarpal fractures were included in a retrospective cohort study. The control group (*n* = 11) underwent a routine recovery, while the treatment group (*n* = 10) received dexamethasone and mannitol injections for five days postoperatively. Serial changes in the degree of pain and fingertip-to-palm distance (FPD) were measured in both groups. The duration from surgery to the initiation of rehabilitation and time to full grip was also compared. *Results:* Compared to the control, the treatment group showed a faster alleviation of pain scores from the postoperative fifth day (2.91 versus 1.80, *p* = 0.013), and faster recovery of FPD from postoperative two weeks (3.27 versus 1.90, *p* = 0.002). Time to physical therapy initiation (6.73 versus 3.80 days, *p* = 0.002) and full grip achievement (42.46 versus 32.70 days, *p* = 0.002) were also faster in the treatment group. *Conclusions:* The steroid-mannitol combination treatment for multiple metacarpal bone fracture patients in the acute postoperative phase promoted the reduction of hand edema and pain, leading to the earlier initiation of physical therapy, rapid improvement in joint motion, and faster achievement of full grip.

## 1. Introduction

Early motion after hand surgery is a crucial factor in postoperative recovery. However, soft tissue edema and pain in the acute postoperative phase are major hurdles for the initiation of rehabilitation. Persistent edema adversely affects the joint range of motion (ROM), soft tissue mobility, and worsens the quality of scar tissue. Pain during rehabilitation also makes the patient reluctant to participate in an active rehabilitation protocol [1]. Eventually, this leads to the suboptimal recovery of joint function and leaves joint stiffness, causing delays to the patient’s return to daily life and work [2]. Despite the importance of postoperative edema and pain control, conventional maneuvers such as elevation, the use of a compression bandage, and massage have all focused on reducing edema using physical force, [1] but these methods are not treatments for the inflammatory mechanism that causes edema itself. This unmet need is especially noticeable in clinical situations such as extensive hand trauma, where severe postoperative edema is expected. Despite the lack of options to reduce postoperative edema, a meaningful method to overcome this important problem is very scarce because postoperative edema is deemed temporary and often overlooked without resorting to active measures in most practices. However, postoperative swelling is a condition that should be actively controlled in the field of hand surgery in order to decrease pain and shorten time to rehabilitation.

Postoperative soft tissue edema is incited due to an acute inflammatory response [3]. As the immune cells and their byproducts accumulate at the injury site, oxidative damage is done to the affected region by reactive oxygen species (ROS). This process increases endothelial permeability and aggravates edema [4]. This cellular interaction is analogous to the pathophysiology of complex regional pain syndrome (CRPS) type 1, in which reactive oxygen free radicals promote an excessive inflammatory response in the neuronal tissue and result in neuropathic symptoms [5]. To reduce this damage, mannitol and steroids are considered as adjunctive treatments from the anti-inflammatory effect of steroids and the free radical scavenging effect of mannitol [6].

Based on this common pathophysiology between postoperative soft tissue edema and CRPS, this study was conceived to see if steroid and mannitol combination treatment could also benefit patients undergoing hand trauma surgery. The patient group was standardized to multiple metacarpal bone fractures to objectively compare the effects of mannitol and steroids among a wide range of hand surgeries. This study aimed to investigate the impact of a steroid and mannitol combination treatment on reducing postoperative edema and pain in patients with multiple metacarpal bones and to determine how it affects the initiation and outcome of rehabilitation treatment.

## 2. Materials and Methods

### 2.1. Patient Selection

This study was approved by the Institutional Review Board of our institution (No. UC22RASE0048, approval date 16 June 2022). All data were analyzed anonymously and according to the principles set forth in the Declaration of Helsinki. This study was conducted through a retrospective chart review of patients who underwent surgery at our institution for closed, unilateral, multiple metacarpal fractures from March 2015 to February 2019. Multiple metacarpal fractures were defined as fractures to two or more metacarpals of the same hand, excluding the thumb metacarpal. Only patients with a closed fracture necessitating reduction and closed pinning with Kirschner wires (K-wires) were included. Exclusion criteria were (1) patients under fifteen years of age; (2) open fracture; (3) open reduction surgery; (4) concomitant trauma in other areas; and (5) any medical condition that contraindicates steroids or mannitol (i.e., hypertension, diabetes, history of peptic ulcer, or electrolyte imbalance, cardiac or renal dysfunction). Consecutive patients who underwent surgery after March 2017 were allocated to the treatment group to which this combination treatment was applied, and patients who had received conventional treatment before that were assigned to the control group. The patient demographics are summarized in Table 1.

### 2.2. Operative Procedure and Study Design

The operation was performed by a single senior surgeon in a uniform fashion (J. Shin). Under general anesthesia or wide-awake local anesthesia no tourniquet (WALANT) preparation, the manual reduction of the fractured metacarpals was performed with fluoroscopy. On confirming acceptable reduction, percutaneous pinning was performed using K-wires. Fixation using K-wires varies from one case to another, but in general, one K-wire is inserted retrogradely and longitudinally into the fractured metacarpal bone, and additional K-wire is transversely inserted into the adjacent unfractured metacarpal bone for the distal segment. The state of functional fixation was checked by passive or active hand grip depending on the type of anesthesia. If displacement of the fractured segment is confirmed during grip, additional fixation is performed. Hand elevation and short-arm splinting under a protective position were applied to all patients.

The control group only received routine postoperative care with nonsteroidal pain relievers (aceclofenac 200 mg twice daily) for seven days. In the treatment group, the slow infusion of 250 mL of 10% mannitol (over one hour) every twelve hours in addition to an 8 mg dexamethasone injection once a day was administered for five consecutive days after surgery, along with the same pain medications in the control group. In general, all patients were treated on an inpatient basis, and were discharged when postoperative pain management was good and rehabilitation education and initiation was successful. However, since the treatment group needed to receive the injection treatment, hospitalization of at least five days was required.

### 2.3. Rehabilitation Protocol

Regardless of the fracture type or severity, the ROM exercise was initiated as soon as passive manual contact between the fingertips and thumb tip was possible. However, if there was resistance due to edema or if the patient complained of pain, it was difficult to expect compliance with the active ROM exercise. These problems were assessed daily, and the rehabilitation treatment was started only when they were resolved. Patients were instructed to follow a standardized rehabilitation program developed at our institution (Table 2). In brief, this protocol was a patient-centered, goal-guided program that focused on incremental ROM recovery using early passive and active exercise to a final active grip-making. The protocol was subdivided into smaller stages (fingertip touch—thumb interphalangeal joint crease touch—thumb metacarpophalangeal joint crease touch—full grip), and was presented to the patient as a goal. The goal was very straightforward, as the instructions were given using the patient’s natural skin crease as the landmark. The same rehabilitation protocol was applied to both groups. Rehabilitation progress was evaluated at weekly intervals with radiologic examination until full grip was achieved. The pins were removed if a full active grip was achieved and there was no radiologic displacement during the rehabilitation period. The splint was kept for two more weeks after pin removal. Afterwards, daily activities were encouraged, but full-strength grip or sports activities were not recommended for at least 3 months.

### 2.4. Outcome Measures

The degree of pain, time to the start of the rehabilitation protocol, the amount of finger ROM recovery, and time to full active grip achievement were evaluated. The degree of postoperative pain was measured with visual analog scale (VAS) scores, and performed by medical personnel unrelated to the study. The measurement was done until seven days after surgery. The amount of finger ROM was measured with a tape ruler in centimeters between the Fingertips to Palmar Crease Distance (FPD) under maximal effort, which is an efficient and reliable method to measure hand ROM [7]. The distance from the tips of the fingertip of each injured ray to the corresponding palmar surface was measured under maximal flexion effort by the patient, and the values were averaged. The measurement was taken by medical personnel unrelated to the study. The FPD was measured every week during the follow-up period. Patients were followed-up for a minimum of eight weeks for the planned measurement.

### 2.5. Statistical Analysis

The collected data was analyzed using the SPSS statistical package software version 24.0 for Windows (SPSS, Chicago, IL, USA). Continuous variables were compared using an independent *t*-test. Categorical variables were compared with Fisher’s exact test and the linear-by-linear association test. All tests were two-sided, and a *p*-value ≤ 0.05 was regarded as significant.

## 3. Results

A total of 21 patients were included in the study according to the inclusion/exclusion criteria. Eleven patients who received only conventional conservative treatment were included in the control group, and 10 patients who received the combination treatment of steroid and mannitol were allocated to the treatment group. The mean age was 32.27 ± 14.21 (range 17–65 years) and 35.20 ± 11.89 (range 18–57 years) in the control group and the treatment group, respectively (*p* = 0.722). The male-to-female ratio was 4.5 and 9 (*p* = 1.000). The causes of trauma were violence, working, slip down, traffic accident, and sports injury (*p* = 0.741). Surgery was performed within 24 h after injury in all patients. The mean time from trauma to surgery was 7.73 ± 2.41 h (range 5–12) in the control group and 6.50 ± 2.32 h (range 4–10) in the treatment group (*p* = 0.250). No patient in either group experienced any postoperative complications.

The multiplicity of fracture was similar in both groups. There were double fractures (nine versus nine) and triple fractures (two versus one), but there were no quadruple fractures (*p* = 1.000). The location of fracture from the second to the fifth ray was also similar in both groups, among which the fourth and fifth ray were the most injured (*p* = 0.575). The anatomical site of the fracture within a metacarpal did not show a significant difference between the two groups, with neck fracture being the most common (*p* = 0.585). In the control group, eight patients underwent surgery under general anesthesia and three underwent surgery while awake. In the treatment group, six patients underwent surgery under general anesthesia and four underwent surgery while awake (*p* = 0.659, Table 1).

**Figure 1:**
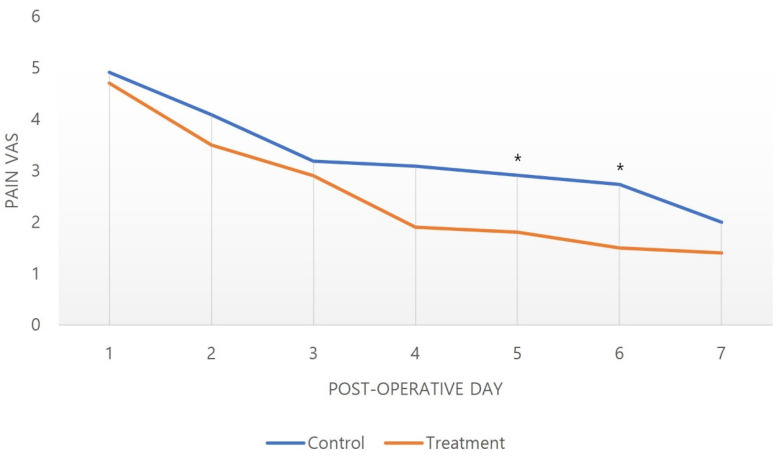
The pain VAS was observed to decrease with time in both groups. However, it was found that there were significant differences on the fifth and sixth day, which means that the pain decreased more rapidly in the treatment group. * *p*-Value ≤ 0.05.

**Figure 2:**
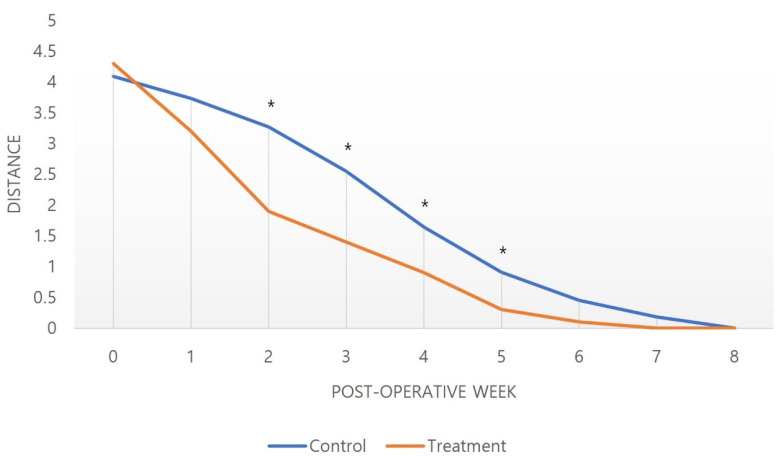
The distance between the fingertip and distal palmar crease (FPD) decreased in both groups over time. However, a significant difference was observed from the second week after surgery, which means that the active flexion was recovering faster in the treatment group. The speed at which the full active grip at which the distance between the fingertip and distal palmar crease became 0 was also achieved more quickly in the treatment group on average. * *p*-Value ≤ 0.05.

Pain VAS scores showed a decreasing trend in both groups. A steeper decline of VAS in the treatment group was noted, which started to indicate statistical significance from the postoperative fifth day (2.91 ± 0.94 versus 1.80 ± 0.92, *p* = 0.013, Figure 1). FPD showed gradual recovery in both groups over time, but a faster decline with statistical significance was found in the treatment group as early as the second postoperative week (3.27 ± 1.10 versus 1.90 ± 0.57 weeks, *p* = 0.002, Figure 2). An earlier start of rehabilitation was possible in the treatment group, which showed statistical significance compared to the control group (6.73 ± 2.15 versus 3.80 ± 1.55 days, *p* = 0.002). Furthermore, a full active grip was accomplished faster in the treatment group, with statistical significance (42.46 ± 6.74 versus 32.70 ± 5.70 days, *p* = 0.002, Table 3).

## 4. Discussion

In patients with hand trauma, early motion accelerates the recovery of the injured joint, enables a quick return to daily life and work, and prevents complications such as joint stiffness [2]. However, postoperative soft tissue edema directly hinders joint excursion in both flexion and extension [1]. Additionally, edema is a significant cause of pain, and is directly related to patient compliance with hand physiotherapy [8,9]. Procedures such as massage, compression, and thermotherapy have been used to treat such initial edema and pain, but their effectiveness is limited [1,10,11,12]. A systematic review by miller et al. that investigated the effectiveness of edema management found a limited benefit and weak confidence to support the routine use of conventional edema therapy other than manual massage [10]. Another systemic review by Ifat et al. concluded that there was some benefit in compression bandaging, but with a strict bandaging protocol [13].

On the other hand, studies that explore a proactive approach to directly inhibit the formation of postoperative swelling and pain are scarce. Therefore, a more direct means of postoperative edema and pain control is still needed to shorten the rehabilitation timeline for extensive hand injuries. In this context, we think that our idea of implementing steroid and mannitol cotreatment in postoperative hand surgery is a feasible attempt to resolve unmet needs.

In hand surgery, steroid and mannitol cotreatment has not been formally investigated in the previous literature. Even recently, studies that explore the therapeutic advantage of steroids and mannitol are focused on neurosurgical and ophthalmological situations [14,15,16,17,18]. Few large-scale meta-analyses have confirmed that mannitol therapy is beneficial for cerebral edema [19,20]. Steroids are also widely known to reverse cerebral edema [21]. For ophthalmologic situations, mannitol and steroid is used in glaucoma treatment [17,18]. A study by Choy et al. investigated the therapeutic potential of 10% mannitol plus steroid cotreatment in CRPS patients, and compared the degree of functional recovery to a group treated with anti-inflammatory agents only. The results showed enhanced recovery in hand function in the mannitol plus steroid group patients, specifically in terms of less pain, better ROM, and higher grip strength. This improvement was seen in over eight months of follow up [22].

The exact mechanism of edema that persists after trauma and surgery is not fully understood. In general, it is known that soft tissue edema of the extremities is caused by a complex inflammatory response such as the accumulation of cytokines and chemokines from neutrophils and macrophages, the extravasation of compliments, and coagulation factors from platelets, bone marrow leakage into peri-fracture tissues, and lymphatic fluid accumulation due to damage of the lymphatic vessels [3]. Another significant factor related to edema and pain aggravation is the ROS is generated from neutrophils [4]. ROS-mediated oxidative stress leads to endothelial dysfunction through the oxidation of cellular signaling proteins, which increases endothelial permeability. This change induces the extravasation of protein-rich fluid through the gap between the endothelial cells, leading to soft tissue edema at the trauma site. This pathophysiology is also a significant concern in CRPS, which is thought to be caused by an excessive inflammatory response involving ROS production [5,6,23].

Focusing on these physiological similarities between the soft tissue microenvironment after surgery and CRPS, we applied a combination of steroid and mannitol treatment used for CRPS to reduce postoperative edema and pain. Steroids are a popular postoperative adjunct in facial and musculoskeletal operations due to the high tendency for edema development in the face and extremities [24,25,26,27,28]. Steroids inhibit the expression of proinflammatory cytokines and the production of inflammatory mediators such as prostaglandins [23]. Although there has been some debate about the actual effect of postoperative edema reduction, the short-term postoperative use of steroids is reported to reduce edema and ecchymosis without increasing the risk of adverse effects [24,27,29].

Mannitol is a hyperosmolar agent and a potent ROS scavenger due to its neutralizing effect of toxic free radicals [5,6,22]. Based on these effects, mannitol is not only an indispensable constituent used for intracranial pressure control, but also used as a neuropathic analgesic for CRPS [6,23]. In general, the mannitol used in CRPS is used at a low dose, and in this case, mannitol acts as an ROS scavenger rather than as an osmotic agent.

In our study, the treatment group that received this combination regimen showed beneficial effects on postoperative recovery and rehabilitation. Pain, measured based on the VAS score, decreased over time in both the control and treatment groups. However, from the fourth day after surgery, the pain in the treatment group started to show better improvement, with a noticeable difference. Statistical significance was indicated on the fifth day after surgery. (Figure 2). This rapid reduction in pain increased patient compliance with rehabilitation, which could advance the initiation of the protocol in the treatment group (Table 3). FPD also showed a tendency to decrease in both groups over time, but showed a statistically significant improvement from the second week in the treatment group. It is thought that the reduction of pain and edema reduced the restriction of joint movement so that the FPD could be rapidly improved along with the rapid start of rehabilitation (Figure 2). In the end, the treatment group achieved a full grip significantly faster (Table 3).

In our trial, no adverse effects related to steroids and mannitol were observed. Steroids may induce adverse effects to the hypothalamic-pituitary axis, in addition to avascular necrosis, postoperative wound infection, the remote possibility of malignancy, diabetes, hyperlipidemia, and peptic ulcer disease [29]. Mannitol may lead to complications such as dehydration, pulmonary congestion, and fluid and electrolyte imbalance [22]. The reason for the absence of side effects is probably due to our low total dosage, slow infusion rate, and distant dosing schedule. Therefore, we think that this regimen can also be implemented as an outpatient-based protocol. However, patient-specific considerations are warranted when co-administering the two agents.

Rehabilitation protocols for closed hand fractures after percutaneous pinning are being performed in various ways by each institute, and an ideal method has yet to be established. However, the initiation of early motion is the common goal of most protocols in order to prevent joint stiffness and limit tendon adhesion. The authors also started active motion exercises as soon as the edema was reduced. In our institute, an easy-to-understand protocol using the natural palmar creases of the thumb has been implemented for metacarpal fractures (Table 2). The main advantage of the proposed protocol is its simplicity. Complex active and passive exercise guidance is naturally incorporated into the protocol. A straightforward visual goal, which is the joint crease of the thumb, is provided to the patient. This protocol also allows for the home-based rehabilitation of patients without medical knowledge, and high patient compliance is easily achieved. The goal is to achieve full grip by six weeks. This method has been used for more than ten years at our institution, and showed safe and satisfactory results.

Several limitations of this study must be acknowledged. First, the degree of edema was not directly measured. Methods for measuring the degree of edema include water volumetry, circumference measurement, or the figure of eight methods [30,31]. However, these methods were not used in the analysis because of the disadvantages of requiring additional equipment, and having significant interpersonal variation between measurements. Second, the number of subjects was small. This is because the inclusion criteria were limited to patients with multiple closed metacarpal fractures who underwent closed reduction and pinning. Hand trauma is very diverse; therefore, it was difficult to objectively analyze the effect of the combination treatment among various severities of trauma. Although additional research is required, we believe that the results of our study will provide one of the alternative methods for various hand trauma patients with severe edema and pain. Third, for the application of steroid-mannitol coadministration in other clinical situations, additional studies are required. The she study was limited to multiple closed metacarpal fractures. The cohort was strictly standardized to unilateral closed, multiple metacarpal fractures for the accurate comparison of postoperative outcomes. However, based on our findings, we think that the indication of our regimen can be expanded and be utilized in other postoperative conditions. Finally, it is unknown how effective this combination is compared to the steroid-only treatment. Steroids are already widely used drugs in hand surgery [25,26]. We borrowed the regimen used in CRPS based on the common pathophysiology of hand edema and CRPS, but to more accurately determine the effect of combining mannitol, there should be a study directly comparing it with a group using only steroids.

## 5. Conclusions

There is still a lack of options to directly combat postoperative edema of the hand. Our data shows that the combination treatment of steroid and mannitol showed positive results in patients with multiple metacarpal fractures, such as rapid pain reduction, the early initiation of rehabilitation exercise, and the rapid recovery of hand function. If there is no contraindication for both drugs, applying this treatment at an early stage after surgery will help patients return to daily life quickly and prevent permanent stiffness. A future study can be directed toward elucidating the exact dosage-response relationship and refining the dosing protocol.

## Figures and Tables

**Table 1:** Summary of patient demographics.

	Control Group (*n* = 11)	Treatment Group (*n* = 10)	*p*-Value
Age (year) *	32.27 ± 14.21	35.20 ± 11.89	
Sex (male to female)	9:2	9:1	0.722
Etiology			1.000
Violence	5	4	0.741
Working	2	3	
Slip down	2	2	
Traffic accident	1	1	
Sports injury	1	0	
Time to surgery (hour) *	7.73 ± 2.41	6.50 ± 2.32	
Multiplicity of fractures			0.250
Double	9	9	1.000
Triple	2	1	
Quadruple	0	0	
Location of fracture(ray) †			0.575
2nd	5	4	
3rd	5	3	
4th	8	7	
5th	6	7	
Anatomic location of fracture †			0.585
Head	2	3	
Neck	12	11	
Shaft	8	5	
Base	2	2	
Anesthesia	2		0.659
General endotracheal anesthesia	8	6	
Wide awake surgery	3	4	

* mean ± standard deviation. † The location and anatomic site of fractures were counted based on the metacarpal bone. As a result, the total number was 24 and 21 in the control and treatment groups, respectively.

**Table 2:** Postoperative rehabilitation protocol for multiple metacarpal fractures in our institution.

Stage	Goal	Protocol *
Stage I	Fingertips to thumb tip by active flexion	-Maintain 10 s of flexion -10 times per cycle -4 cycles per day
Stage II	Fingertips to thumb interphalangeal joint crease by active flexion	
Stage III	Fingertips to thumb metacarpophalangeal joint crease by active flexion	
Stage IV	Fingertips to distal palmar crease by active flexion	

* The rehabilitation program was initiated immediately when passive fingertips to thumb tip contact becomes possible. A splint is always applied when not in physiotherapy. If active flexion is impossible, passive flexion with the uninjured hand was attempted. If the goal was not achieved, it was repeated until assessment the following week.

**Table 3:** Summary of the start time of rehabilitation and the time of achieving a full active grip in both groups.

	Control Group (*n* = 11)	Treatment Group (*n* = 10)	*p*-Value
Rehabilitation initiation (days)	6.73 ± 2.15	3.80 ± 1.55	0.002 *
Full active grip (days)	42.46 ± 6.74	32.70 ± 5.70	0.002 *

* *p*-Value ≤ 0.05.

## Data Availability

The data presented in this study are available on request from the corresponding author.
